# Using feasibility dose–volume histograms to reduce intercampus plan quality variability for head‐and‐neck cancer

**DOI:** 10.1002/acm2.13749

**Published:** 2022-08-12

**Authors:** Saeed Ahmed, Chieh‐Wen Liu, Danielle LaHurd, Eric Murray, Matthew Kolar, Nikhil Joshi, Neil Woody, Shlomo Koyfman, Ping Xia

**Affiliations:** ^1^ Department of Radiation Oncology, Taussig Cancer Center Cleveland Clinic Foundation Cleveland Ohio USA; ^2^ Department of Radiation Oncology Rush University Medical Center Chicago Illinois USA

**Keywords:** auto‐planning, feasibility DVH predictions, head‐and‐neck cancer, plan quality variability

## Abstract

The purpose of this work is to objectively assess variability of intercampus plan quality for head‐and‐neck (HN) cancer and to test utility of a priori feasibility dose–volume histograms (FDVHs) as planning dose goals. In this study, 109 plans treated from 2017 to 2019 were selected, with 52 from the main campus and 57 from various regional centers. For each patient, the planning computed tomography images and contours were imported into a commercial program to generate FDVHs with a feasibility value (*f*‐value) ranging from 0.0 to 0.5. For 10 selected organs‐at‐risk (OARs), we used the Dice similarity coefficient (DSC) to quantify the overlaps between FDVH and clinically achieved DVH of each OAR and determined the *f*‐value associated with the maximum DSC (labeled as *f*‐max). Subsequently, 10 HN plans from the regional centers were replanned with planning dose goals guided by FDVHs. The clinical and feasibility‐guided auto‐planning (FgAP) plans were evaluated using our institutional criteria. Among plans from the main campus and regional centers, the median *f*‐max values were statistically significantly different (*p* < 0.05) for all OARs except for the left parotid (*p* = 0.622), oral cavity (*p* = 0.057), and mandible (*p* = 0.237). For the 10 FgAP plans, the median values of *f*‐max were 0.21, compared to 0.37 from the clinical plans. With comparable dose coverage to the tumor volumes, the significant differences (*p* < 0.05) in the median *f*‐max and corresponding dose reduction (shown in parenthesis) for the spinal cord, larynx, supraglottis, trachea, and esophagus were 0.27 (8.5 Gy), 0.3 (7.6 Gy), 0.19 (5.9 Gy), 0.19 (8.9 Gy), and 0.12 (4.0 Gy), respectively. In conclusion, the FDVH prediction is an objective quality assurance tool to evaluate the intercampus plan variability. This tool can also provide guideline in planning dose goals to further improve plan quality.

## 1 INTRODUCTION

Head‐and‐neck (HN) cancer is associated with considerable morbidity and mortality with an annual incidence of ∼4% and an annual death rate of 2.4% in the United States.^1^, ^2^ The tumor volumes of HN cancer are surrounded by a multitude of serial and parallel organs, making treatment planning a challenging task in radiotherapy.

Inverse planning in intensity‐modulated radiotherapy (IMRT) is an iterative and labor‐intensive process. With multiple surrounding organs‐at‐risk (OARs), treatment plan qualities for HN cancer may vary drastically, depending on the clinical workload, available resources, and experiences of planners. In a well‐controlled study, Nelms et al. attributed the plan quality variations to inter‐planner skill and experience.^3^ Many other investigators have explored the variability in treatment plan quality among planners^3^, ^4^, ^5^, ^6^, ^7^ and in different institutions.^8^, ^9^ Recently, many major cancer centers have expanded their services through regional centers to offer convenience to patients while improving the quality of patient care. Thus, many efforts are devoted to standardize clinical practices and to reduce variability in radiotherapy planning. One approach to standardize treatment planning quality and practice is to provide planners with specific planning dose guidelines and continuous training. The other approach is to use automation and predictive models to minimize the human intervention during treatment plan optimization.^10^ Different automation methods, such as multi‐criteria optimization,^11^, ^12^ knowledge‐based planning (KBP),^13^, ^14^, ^15^ template‐based auto‐planning,^16^, ^17^ and a priori feasibility dose–volume histogram (FDVH) prediction,^18^ are incorporated in major treatment planning systems (TPSs) and have been evaluated with HN cases. The plan qualities produced from these automation methods were reported to be comparable.^19^, ^20^, ^21^

PlanIQ (Sun Nuclear Corporation, Melbourne, FL.), an FDVH prediction tool has been integrated into the Pinnacle TPS v. 16.2 (Philips Healthcare Inc., Cleveland, OH) recently. The patient‐specific FDVH predictions can guide planners to achieve a clinically feasible plan without using a trial‐and‐error process to search for achievable planning dose objectives. PlanIQ also offers an objective plan quality metric for plan evaluation. The auto‐planning module in Pinnacle TPS has been investigated,^16^, ^17^, ^22^ but there were very limited studies on the feasibility‐guided auto‐planning (FgAP) for HN cases. Cilla et al.^23^ evaluated an FDVH‐guided personalized planning for 15 locally advanced HN cancer patients. The authors found that the FgAP plans were superior to the manual plans in dose conformity to the tumor targets and in normal tissue sparing. The FgAP method further improved planning efficiency by reducing the planning time to less than 30 min. Perumal et al.^24^ studied the FgAP plan quality with one case from several different sites and evaluated plans according to Radiation Therapy Oncology Group (RTOG) criteria. For the HN case, they reported that improved OAR sparing was observed with the FgAP. Alves et al.^25^ showed that feasibility predictions were particularly useful for sparing the serial organs (spinal cord and brainstem) of HN plans in helical tomotherapy. Xia et al.^26^ studied the FgAP for lung cancer and found that FgAP plans improved plan quality with reduced lung dose.

The purpose of this study was twofold: (1) to assess our intercampus variability in plan quality for HN cancer using the feasibility prediction tool, and (2) to investigate whether the plan quality under the FDVH‐guided auto‐planning can be improved for selected HN cases.

## 2 METHODS

### 2.1 Plan selection

From an institutional review board approved registry, we retrospectively selected 109 patients with HN cancer from the main and regional centers in our network treated from 2017 to 2019. Primary cancer sites for these patients were oropharynx, nasopharynx, and hypopharynx treated with bilateral nodal volumes. All selected plans had a definitive treatment intent. All plans had the same prescription dose of 70 Gy to the planning target volume (PTV) of gross tumors and 56 Gy to PTVs of the elective nodal volumes. The distributions of specific disease sites for these patients are listed in Table 1. The median volumes of the primary tumors and neck nodes for these patients were 110 and 137.4 cm^3^, respectively. All cases were planned with volume‐modulated arc therapy (VMAT) using simultaneous integrated boost technique with three full arcs and treated on Varian TrueBeam machines equipped with either standard or high‐definition multi‐leaf collimators (Varian Medical Systems, Palo Alto, CA). The collimator angles were set at ±10° for the first two arcs and 90° for the third arc. The dose was calculated with an isotropic dose calculation grid size of 4 mm with a 4° control point spacing.

**TABLE 1:** Percentage of the patients selected from head‐and‐neck (HN) disease sites

		Percentage of patients		
No.	Disease site	Main (52) (%)	Regions (57) (%)	Total (109) (%)
1	Nasopharynx	7.7	8.8	8.2
2	Oropharynx	80.7	66.6	73.4
3	Hypopharynx	–	3.5	1.8
4	Larynx	11.5	21	16.5

### 2.2 FDVH predictions

The algorithm of FDVH predicts dose outside the PTVs using empirical high gradient dose spread (HGDS) functions to account for the effect of penumbra and low‐dose spread (LDS) kernels to account for beam attenuation and out‐of‐field scattering while assuming full dose coverage to the PTV. The details of the algorithm were described by Ahmed et al.^18^ Briefly, the FDVH algorithm considers the anatomic relationship between the planning organs‐at‐risk and OARs according to patient‐specific planning computed tomography (CT) images. Using the HGDS functions and LDS kernels, a benchmark dose distribution surrounding the PTV is generated. From the bounding voxels of the PTV, the radiological distances to each voxel in the dose cloud is calculated based on the density information from the planning CT images. Finally, the FDVH for each organ is estimated from the benchmark dose with a feasibility value (*f*‐value) ranging from 0 to 1. The dose estimated to the PTV bounding voxels generates the feasibility curve with *f*‐value = 0. The area under this curve is labeled the impossible region. The FDVHs with higher *f*‐values (>0) are estimated by the normalized distance from the zero FDVH curve (*f*‐value = 0) in the dose–volume space, thereby generating three more feasibility regions labeled as the difficult region with *f*‐values ranging from 0 to 0.1, the challenging region with *f*‐values ranging from 0.1 to 0.5, and the probable region with *f*‐values ranging from 0.5 to 1.

### 2.3 Assessment of variability in plan quality

**FIGURE 1:**
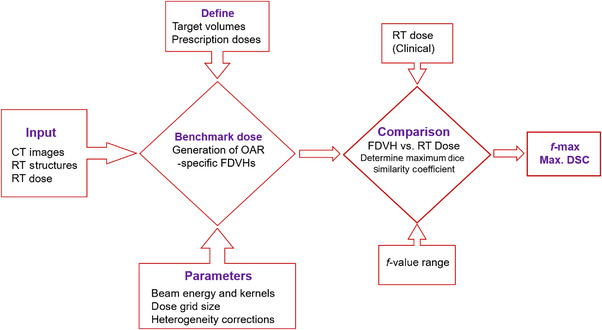
Flowchart illustrating the procedure for the assessment of variability in plan quality

The FDVH can be used as an objective measure to assess the variability in plan quality. In this study, PlanIQ (v. 2.2) was used, running on a standalone workstation. For each patient, the planning CT images, RT structures, RT plan, and RT dose were transferred from our TPS to PlanIQ workstation. The benchmark dose for each plan was calculated using the treatment beam energy with a 3‐mm isotropic dose calculation grid size. The method and workflow are illustrated in Figure 1. The selected OARs for FDVHs included the spinal cord, parotids, oral cavity, supraglottis, mandible, constrictors, trachea, esophagus, and larynx. The FDVHs for these OARs were generated with an *f*‐value in a range of 0–0.5 at 0.01 intervals. The generated FDVHs were compared with the clinical DVHs using the Dice similarity coefficient (DSC) as defined in the following equation:

(1) $$DSC\;=\;2\frac{A\;\cap \;B}{A\;+\;B}$$

where *A* and *B* are areas under the clinical DVH and FDVH curves, respectively, and *A* ∩ *B* is the intersection of areas. The DSC was calculated using a built‐in routine in the PlanIQ program. To evaluate the clinical DVH of each OAR, the *f*‐value associated with the maximum DSC was determined and labeled as *f*‐max. The smaller *f*‐max of an OAR indicated that the clinical plan achieved better OAR sparing when compared with the other clinical plans.

### 2.4 Using FDVHs to guide auto‐planning

From the regional centers, 10 plans that had a higher *f*‐max than that of main campus plans were selected for FDVH‐guided replanning using the auto‐planning module of Pinnacle TPS v. 16.2. These plans are referred to as FgAP plans. The FgAP plans used same dose prescription and planning technique as those of the original clinical plans described in Section 2.1. The dose falloff was controlled by two dose rings created by subtracting 1‐ and 3‐cm expansions of the combined PTVs from the patient's external contour.

The FDVH‐predicted dose objectives were input to the auto‐planning optimizer as the dose limiting goals for all OARs. Table 2 lists the typical FDVH‐guided planning objectives, ranges of the *f*‐value, and planning objectives for two added dose shaping rings. The functionality of auto‐planning module and optimization process were described previously.^16^, ^17^, ^22^ The auto‐planning module requires users to define parameters in the advanced page. In this work, we used the treatment technique described by Gintz et al.^16^ with slight modifications in the advanced settings. The tuning balance, dose falloff margin, and hot‐spot maximum goal were set at “10,” 1 cm, and 105%, respectively. The use of cold‐spot regions of interest was allowed. After the first round of auto‐planning, the planning objectives used in the auto‐planning module were listed in the conventional optimization module, allowing further optimization. After optimization, two to three additional conventional optimizations were performed in this study, each with 30 iterations, to further improve the target coverage to the PTVs while reducing the maximum dose of the plan.

**TABLE 2:** An example of organ‐at‐risk optimization objectives used in auto‐planning for the feasibility‐guided replans

**OAR**	**Objective type**	**Dose (cGy)**	**Volume**	**Priority**	**Compromise**	***f*‐Value range**
HD–PTV^a^	Min dose	7000				
LD–PTV^a^	Min dose	5600				
Ring 1 cm	Max dose	3500		Low	Yes	–
Ring 3 cm	Max dose	2100		Medium	Yes	–
Brainstem	Max dose	2500		High	No	0.05–0.15
Brainstem + PRV3	Max dose	3500		High	No	0.1
Spinal cord	Max dose	3500		High	No	0.1–0.2
Spinal cord + PRV5	Max dose	4000		High	No	0.1–0.2
Parotids	Mean dose	2600		Medium	Yes	0.05–0.15
Larynx	Mean dose	3000		Medium	Yes	0.05–0.2
Supraglottis	Mean dose	4500		Medium	Yes	0.05–0.2
Esophagus	Mean dose	2500		Medium	Yes	0.05–0.2
Trachea	Mean dose	2500		Medium	Yes	0.05–0.2
Lips	Mean dose	600		Medium	Yes	0.1–0.2
Oral cavity	Mean dose	3000		Medium	Yes	0.1–0.25
Constrictors	Mean dose	4500		Medium	Yes	0.1–0.25
Submandibular	Mean dose	3800		Medium	Yes	0.1–0.2
Mandible	Max DVH	7300	1 cm^3^	Medium	Yes	0.1–0.25
Eye lenses	Max dose	170		Medium	Yes	–
Brachial plexus	Max dose	6300		High	Yes	–

*Note*: The last column describes the FDVH *f*‐values used for the optimization objectives.

Abbreviations: DVH, dose–volume histogram; FDVH, feasibility dose–volume histogram; OAR, organ‐at‐risk.

^a^HD–PTV, high dose–planning target volume; LD–PTV, low dose–planning target volume.

#### 2.4.1 Plan evaluation

The clinical and FgAP plans were evaluated using our institutional established criteria for HN cancer. All plans were normalized such that 95% of PTVs received the corresponding prescription doses. The maximum dose of an OAR was defined as the dose encompassing a voxel of 0.03 cm^3^ of the OAR. The evaluation criteria are listed in Table 3. For high dose–PTVs (HD–PTVs), RTOG conformity index (CI) and homogeneity index (HI), defined in the following equations, were calculated:

(2) $$CI\;=\frac{V_{Rx}}{V_{PTV}}$$

(3) $$HI\;=\frac{D_{max}}{D_{Rx}}\;$$

where $V_{Rx}$ is the volume covered by the HD–PTV prescription isodose line, $V_{PTV}$ is the volume of HD–PTV, $D_{max}$ is the maximum dose to the HD–PTV, and $D_{Rx}$ is the prescription dose (70 Gy). The *f*‐values corresponding to the maximum DSC (*f*‐max) for both clinical and FgAP plans were determined as described in Section 2.3. The smaller *f*‐max indicates improved OAR sparing and better plan quality, provided that the PTVs are adequately covered with the prescription dose as defined in the evaluation criteria.

**TABLE 3:** Median values of dosimetry metrics and *f*‐values for the clinical and feasibility‐guided auto‐planning (FgAP) plans

OAR	Dose value	Evaluation criteria	Dosimetric analysis			*f*‐max		
			Clinical	FgAP	*p*‐Value	Clinical	FgAP	*p*‐Value
HD–PTV	*V*_70 Gy_ (%)	≥95%	95.3(1.3)	95.3(0.4)	0.96	–	–	–
	*V*_73.5 Gy_ (%)	≤115%	14.7(8.9)	13.8(5.3)	0.33	–	–	–
	$D_{0.03\;cm^{3}}$	≤77 Gy	75.3(0.6)	76.7(1.8)	0.05	–	–	–
	CI	–	1.14(0.12)	1.12(0.14)	0.08	–	–	–
	HI	–	1.09(0.01)	1.11(0.02)	0.04	–	–	–
LD–PTV	*V*_56 Gy_ (%)	≥95%	95.9(2.7)	96.9(2.0)	0.58	–	–	–
Brainstem	$D_{0.03\;cm^{3}}$	<25 Gy	23.0(7.3)	16.6(7.2)	0.007	0.05(0.06)	0.04(0.03)	0.027
Brainstem_PRV3	$D_{0.03\;cm^{3}}$	<35 Gy	29.3(7.7)	20.6(14.3)	0.022	–	–	–
Spinal cord	$D_{0.03\;cm^{3}}$	<35 Gy	30.8(6.0)	22.3(4.1)	0.005	0.47(0.09)	0.20(0.08)	0.005
Spinal cord_PRV5	$D_{0.03\;cm^{3}}$	<38 Gy	38.6(7.8)	37.6(14.6)	0.114	–	–	–
Parotid right	*D_mean_*	<26 Gy	24.1(7.1)	22.3(10.3)	0.241	0.12(0.19)	0.07(0.11)	0.043
Parotid left	*D_mean_*	<26 Gy	22.3(7.0)	19.4(3.6)	0.169	0.27(0.15)	0.18(0.13)	0.261
Mandible	$D_{0.03\;cm^{3}}$	<75 Gy	72.9(1.1)	72.5(1.5)	0.878	0.46(0.09)	0.46(0.15)	0.141
Oral cavity	*D_mean_*	<30 Gy	28.6(11.8)	29.5(11.0)	0.386	0.36(0.1)	0.40(0.09)	0.475
Lips	*D_mean_*	<7 Gy	6.4(2.6)	6.0(0.9)	0.11	–	–	–
Constrictors	*D_mean_*	<45 Gy	53.7(6.5)	52.3(6.7)	0.007	0.49(0.14)	0.44(0.19)	0.293
Supraglottis	*D_mean_*	<45 Gy	43.5(9.4)	37.6(15.5)	0.008	0.47(0.2)	0.28(0.19)	0.014
Larynx	*D_mean_*	<30 Gy	26.5(10.3)	18.9(8.0)	0.007	0.48(0.08)	0.18(0.09)	0.018
Trachea	*D_mean_*	<25 Gy	24.0(10.4)	15.1(12.0)	0.005	0.42(0.15)	0.23(0.09)	0.011
Esophagus	*D_mean_*	<25 Gy	21.2(11.1)	17.3(6.7)	0.005	0.33(0.2)	0.21(0.08)	0.013
Thyroid	*D_mean_*	–	48.7(14.4)	41.5(15.1)	0.012	0.26(0.21)	0.14(0.14)	0.008
Submandibular right	*D_mean_*	<39 Gy	54.7(14.4)	46.3(18.5)	0.31	–	–	–
Submandibular left	*D_mean_*	<39 Gy	37.6(4.4)	38.0(11.0)	0.508	–	–	–

*Note*: IQRs for each parameter are given in parentheses.

Abbreviations: CI, conformity index; FgAP, feasibility‐guided auto‐planning; HD–PTV, high dose–planning target volume; HI, homogeneity index; IQRs, Interquartile ranges; LD–PTV, low dose–planning target volume; OAR, organ‐at‐risk.

For comparison of plan quality between the clinical and FgAP plans, the median values of the evaluated OAR dose metric ($D_{0.03\;cm^{3}}$ or *D_mean_*) and *f*‐max were reported. Furthermore, for each selected OAR, the difference of *f*‐max between the clinical and FgAP plan, defined as *(∆f*‐max *= f*‐max*_clinical_–f*‐max*_FgAP_*), was calculated and so was the corresponding dose change.

### 2.5 Patient‐specific quality assurance (PSQA)

For 8 of 10 selected plans, the patient‐specific quality assurance (PSQA) was performed for both clinical and FgAP plans using an electronic portal imaging device and PerFRACTION dose verification system (Sun Nuclear Corporation, Melbourne, FL.) with the fraction zero absolute dose mode. The Digital Imaging and Communication in Medicine data for all 10 plans were archived but the original plans for two patients were lost, which were necessary for QA. The measured and planned dose distributions for each beam were compared using 2D gamma analysis using three metrics of 2%/2, 3%/2, and 3%/3 mm with the global normalization and 10% low‐dose threshold. The passing criterion was set at 95%. The difference in the point dose between the clinical and FgAP plans for each beam was reported, and the monitor units (MUs) from these plans were compared.

### 2.6 Statistical analysis

The statistical analysis was performed using SPSS v. 24 (IBM Inc., Armonk, NY). The distribution of *f*‐max values of analyzed OARs did not follow a normal distribution. Therefore, we used the Mann–Whitney *U* test to evaluate plan quality variability between the main campus and the regional centers. To compare the clinical plans and FgAP plans, we used the Wilcoxon signed‐rank test to determine the statistical significance in the median dose and median *f*‐max of studied OARs. The significance level was set at 0.05.

## 3 RESULTS

### 3.1 Plan variability

**FIGURE 2:**
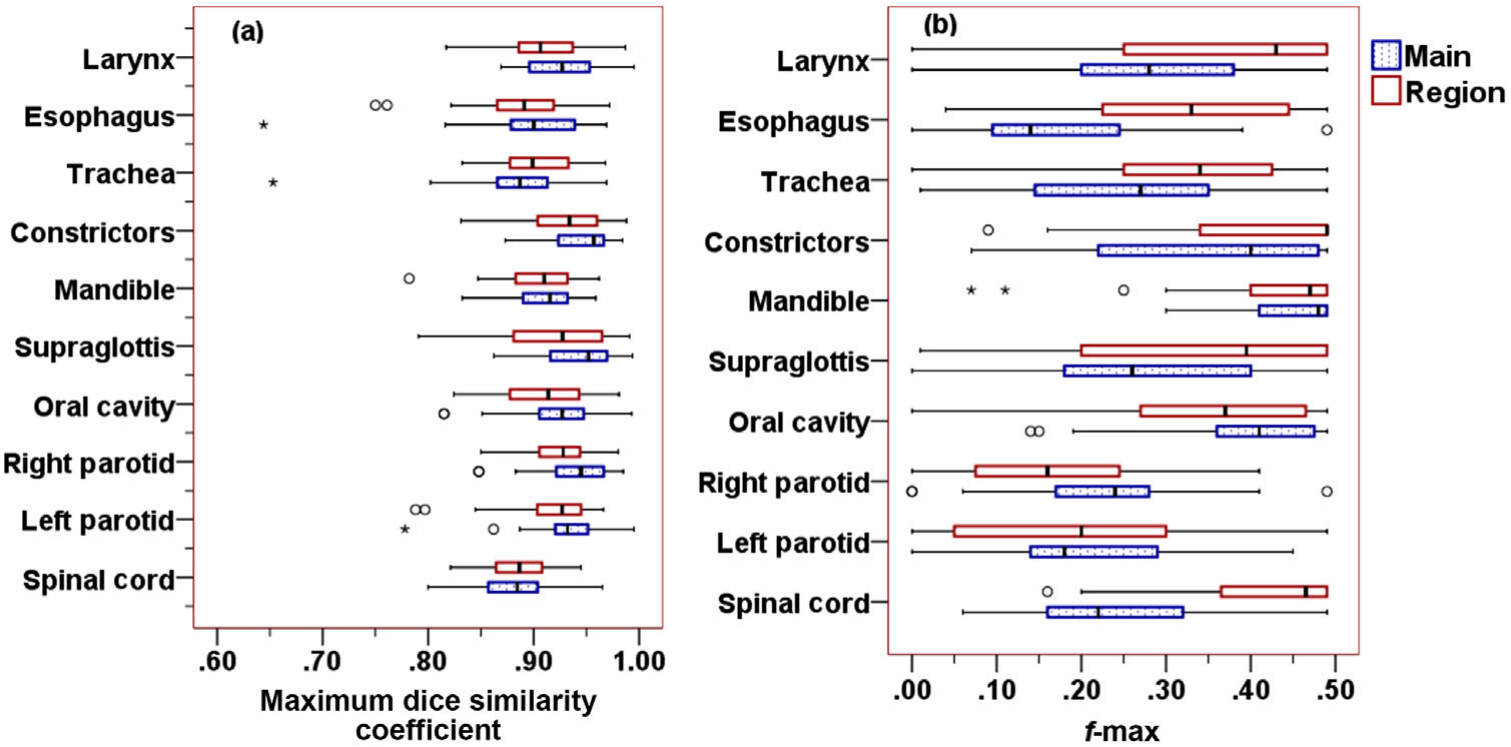
Comparison of maximum Dice similarity coefficients (a) and *f*‐max values (b) for all organs‐at‐risk (OARs) between the head‐and‐neck (HN) treatment plans from the main campus and regional centers

The maximum DSCs of FDVHs and clinical DVHs of selected 10 OARs from the main and regional plans are shown in Figure 2(a). A larger DSC value of the OAR means a greater overlap between the clinical DVH and FDVH. The median values of the maximum DSC and the interquartile ranges (IQRs) of DSC for all OARs were similar for the main and regional plans. The largest DSC values were 0.96 and 0.93 for the constrictors from the main campus plans and regional plans, respectively. The smallest DSC was 0.89 for the spinal cord from both main campus and regional plans. Although the maximum DSCs were comparable for the main and regional plans, large differences in the *f*‐max values of OARs were observed.

Figure 2(b) compares the *f*‐max of DVHs from the main campus and regional plans for 10 selected OARs. The overall median values (and IQR) of *f*‐max for the main and regional plans were 0.28 (IQR = 0.24) and 0.36 (IQR = 0.27), respectively. The median values of *f*‐max of all OARs for the main campus plans were smaller than those of the regional plans, except for the median values of right parotid (0.24 vs. 0.16), oral cavity (0.41 vs. 0.37), and mandible (0.48 vs. 0.47). When comparing *f*‐max values from the main campus plans and regional plans, the differences were statistically significant (*p* < 0.05) for all OARs except for the left parotid (*p* = 0.611), oral cavity (*p* = 0.15), and mandible (*p* = 0.453). The IQRs for the main campus plans were smaller than those of the regional plans for all OARs, except for the IQRs of the spinal cord (0.16 vs. 0.12) and constrictors (0.26 vs. 0.15), indicating that plan quality variability existed among our centers. As shown in Figure 2(b), the smallest IQRs were of the mandible from both main campus and regional plans, indicating a consistency of sparing the mandible among our centers. For plans from the main campus, a median *f*‐max ≥ 0.4 was associated with the mandible (0.48), oral cavity (0.41), and constrictors (0.40), whereas the esophagus had the smallest median *f*‐max of 0.14. For plans from the regional centers, a median *f*‐max ≥ 0.4 was associated with the constrictors (0.49), mandible (0.47), spinal cord (0.47), larynx (0.43), and supraglottis (0.4), whereas the smaller values of the median *f*‐max were 0.2 for the left parotid gland, and 0.16 for the right parotid gland. As shown in Figure 2(b), the parotid glands were preferentially spared more than the other OARs among the main and regional centers. Differences in *f*‐max between the plans from the main and reginal centers were noticeable for OARs located medially such as the spinal cord, larynx, trachea, and esophagus.

### 3.2 Feasibility‐guided replanning

The dosimetric comparison between the clinical and FgAP plans is summarized in Table 3. For both clinical and FgAP plans, 95% of the PTVs received the respective prescription doses. When compared to the clinical plans, the dose coverage of the low dose–PTV (LD–PTV) was slightly better for FgAP plans with no statistical difference. The hotspot ($D_{0.03\;cm^{3}}$) within the PTV was greater for the FgAP plans (the median $D_{0.03\;cm^{3}}$ 76.6 vs. 75.3 Gy), but the median *V*_73.5 Gy_ (%) was smaller for the FgAP plans than that of the clinical plans. The CIs of FgAP plans were better than those of the clinical plans. The HIs were better for the clinical plans. The differences in the median values of all PTV‐related dosimetric metrics were not statistically significant (*p* > 0.05) except for the HIs.

**FIGURE 3:**
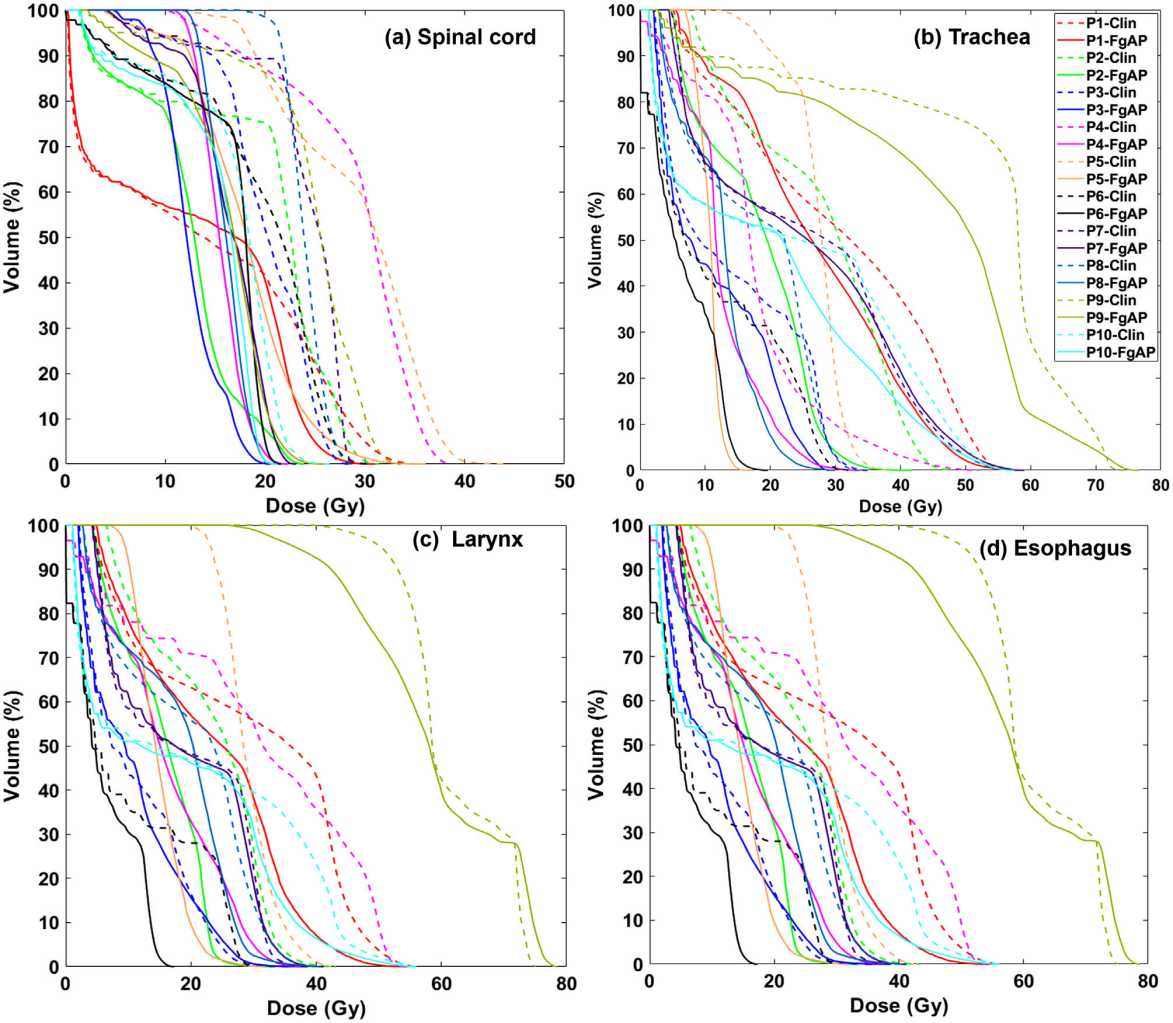
Comparison of dose–volume histograms (DVHs) between feasibility‐guided auto‐planning (FgAP) plans and clinical plans for spinal cord (a), trachea (b), larynx (c), and esophagus (d). The figure shows the DVHs from all patients.

The median values of OAR dosimetric metrics and the *f*‐max for FgAP plans were similar to or smaller than those of clinical plans. The median dose differences were statistically significant (*p* < 0.05) for the OARs located medially, such as the brainstem (6.4 Gy), spinal cord (8.5 Gy), supraglottis (5.9 Gy), larynx (7.6 Gy), esophagus (3.9 Gy), trachea (8.9 Gy), and thyroid (7.2 Gy). The median values of the maximum dose ($D_{0.03\;cm^{3}}$) to the mandible and mean dose (*D_mean_*) to the parotid glands, oral cavity, lips, and submandibular glands for FgAP plans were comparable to those of the clinical plans without statistically significant differences. For these 10 cases, DVH comparison between the FgAP and clinical plans for four medially located OARs (the spinal cord, larynx, trachea, and esophagus) identified from Figure 2(b) are shown in Figure 3.

**FIGURE 4:**
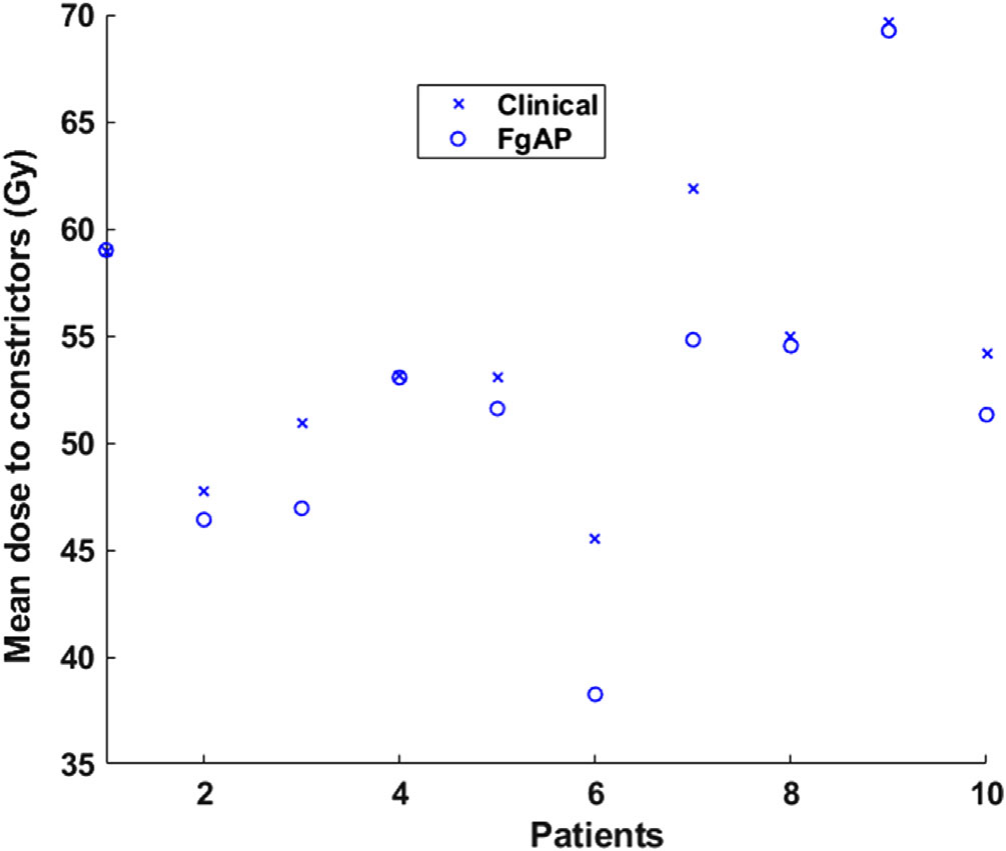
Comparison of the mean dose to the constrictors for feasibility‐guided auto‐planning (FgAP) and clinical plans

**FIGURE 5:**
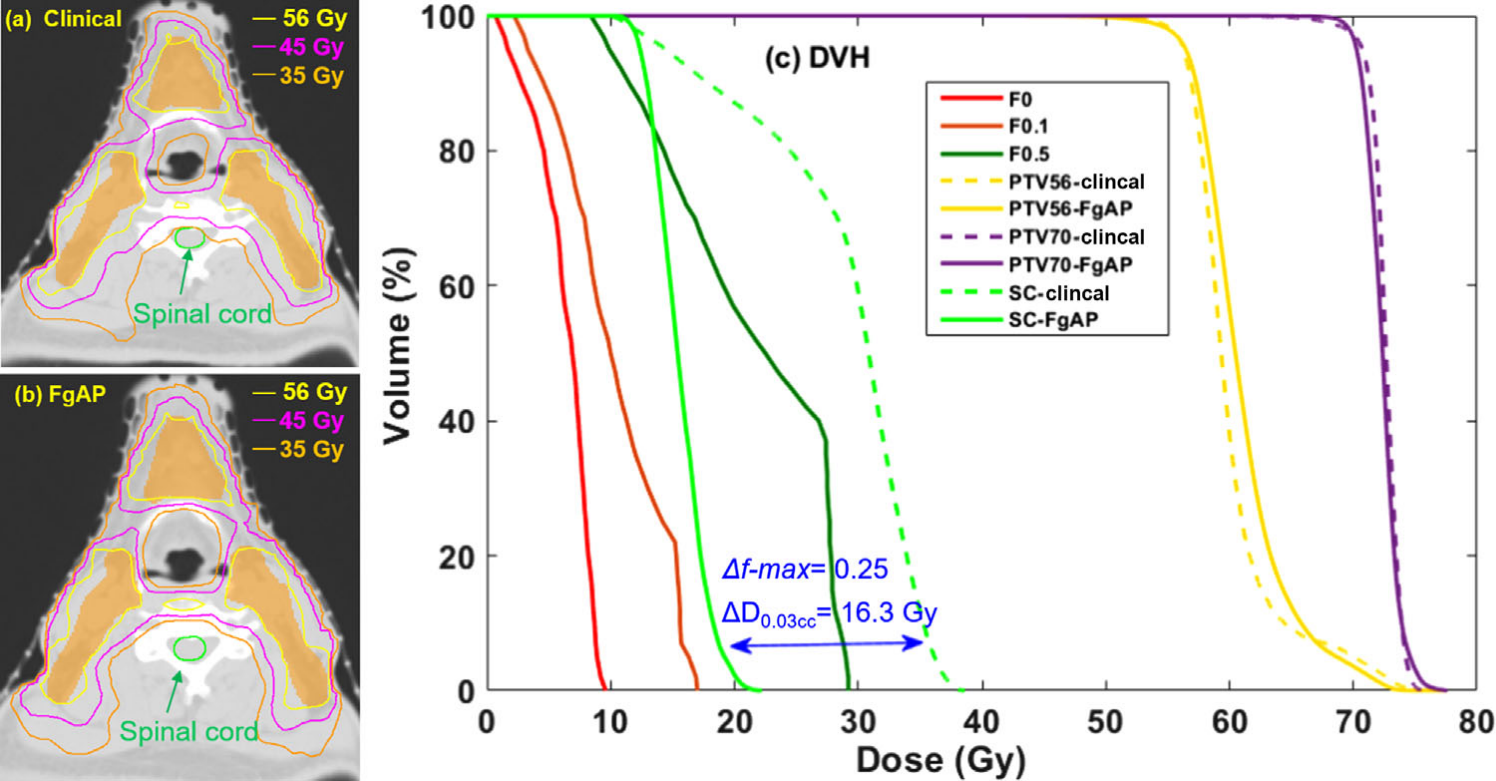
An example of a spinal cord sparing plan for a nasopharynx case. The low dose–planning target volume (LD–PTV) in solid light orange, the spinal cord in light green, and isodose lines are displayed in an axial image from the clinical plan (a) and from the feasibility‐guided auto‐planning (FgAP) plan (b). The panel (c) displays dose–volume histograms (DVHs) of the targets and spinal cord from the FgAP and clinical plans as well as the feasibility dose–volume histograms (FDVHs) for the spinal cord at different feasibility levels.

The median *f*‐max values from FgAP and clinical plans are listed in the last two columns of Table 3. The median *f*‐max values were similar or smaller for FgAP plans when compared to those of the clinical plans. Large‐to‐moderate differences (Δ*f = f_clinical_–f_FgAP_*) of the median *f*‐max values were observed in the spinal cord (0.27), right parotid (0.05), supraglottis (0.19), larynx (0.3), trachea (0.19), esophagus (0.12), and thyroid (0.12). For constrictors, the median dose difference between the FgAP and clinical plans was statistically significant (*p* = 0.007), whereas the difference in the median *f*‐max values was not (*p* = 0.29). The mean dose to constrictors for all plans is plotted in Figure 4, which shows that the dose to the constrictors in FgAP plans was similar to or smaller than those of clinical plans. Example isodose distributions and associated DVHs from the clinical and FgAP plans for patient dataset 4 are shown in Figure 5. As shown in Figure 5, the 35‐Gy isodose line (50% of prescription dose) in FgAP plans is pushed away from the spinal cord and resultant DVH of the spinal cord is approaching to the difficult region of FDVH. The $D_{0.03\;cm^{3}}$ of the spinal cord was 22 Gy for the FgAP plan and 38.3 Gy for the clinical plan with a Δ*f* of 0.25.

**FIGURE 6:**
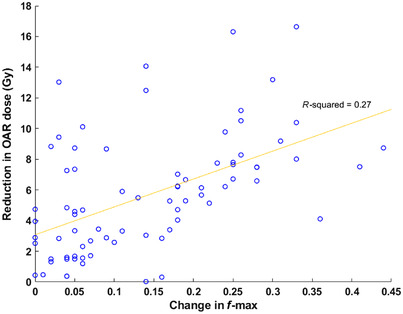
The organ‐at‐risk (OAR) dose reduction with the change in the *f*‐max values

For all OARs, the FgAP plans achieved a median *f*‐max value similar to the plans from the main campus (cf Section 3.1 and Figure 2), indicating the potential of FgAP to reduce intercampus plan quality variability. Figure 6 shows an individual OAR dose reduction (Δ*D = D_clinical_–D_FgAP_)* as a function of change in *f*‐max values (Δ*f*) for all FgAP plans. From Figure 6, it is shown that Δ*D* > 2 Gy for most of the OARs with a Δ*D_max_* exceeding 16 Gy. For the Δ*f* > 0.05, a linear relationship between Δ*D* and Δ*f* may be observed. Large variations from the linear behavior were observed for Δ*f* ≤ 0.05 where small change in *f*‐max values resulted in large dose reduction to OARs. For all OARs, the dose reductions to the OARs were correlated with the corresponding decrease in *f*‐max for each OAR; however, a weak correlation (*R* ^2^ = 0.27) was observed.

### 3.3 PSQA

**FIGURE 7:**
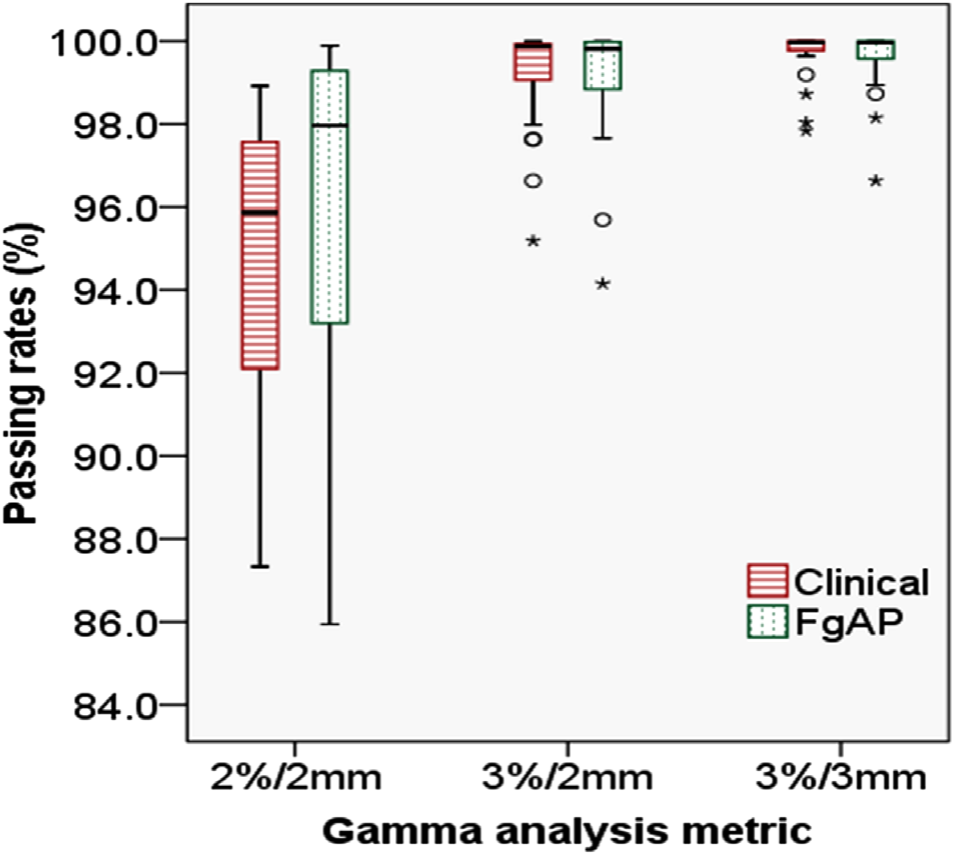
Comparison of gamma passing rates between the clinical and feasibility‐guided auto‐planning (FgAP) plans

On average, MUs from FgAP plans were greater than those from clinical plans, indicating an increased modulation in FgAP plans. The average MUs per fraction for FgAP plans and clinical plans were 904 ± 142 (range 709–1142) and 772 ± 126 (range 625–969), respectively. The passing rates for all gamma indices are plotted in Figure 7. The average passing rates at the American Association of Physicists in Medicine Task Group‐218 recommended gamma metric (3%/2 mm with global normalization and 10% low‐dose threshold) were 99.1% ± 1.5% and 99.2% ± 1.3% for FgAP and clinical plans, respectively. The average passing rates for the other two gamma metrics (2%/2 and 3%/3 mm) were ≥95% for both FgAP and clinical plans. The passing rates at all gamma metrics for FgAP plans were similar to those of the clinical plans (*p* > 0.05). The average percent point dose differences between calculation and measurement for FgAP plans and clinical plans were 0.3% ± 1.3% and −0.2% ± 1.4%, respectively.

## 4 DISCUSSION

The purpose of this study was to explore intercampus plan quality variability for HN cancer. It should be noted that our planners at the main campus were more experienced in HN treatment planning than those of the regional centers, because the number of HN cancer patients treated at the main campus is greater than that at each regional center. Using the FDVH as an objective QA tool to evaluate plan quality, we found large differences in HN planning practices between the main campus and regional centers, particularly for the OARs located medially, such as the spinal cord, larynx, supraglottis, trachea, and esophagus. The larger *f*‐max values of these OARs from regional plans indicated an opportunity of further improvement in plan quality. Interestingly, the planners from both main campus and regional centers worked hard to spare the parotid glands, demonstrated by the similar small *f*‐max values. Hazel et al.^17^ found that the variability among planners existed even at centers with experience in IMRT planning and the inter‐planner variation is attributed to a short planning time and lack of patient anatomic specific predictions of achievable dose goals in optimization. Scaggion et al.^4^ demonstrated plan quality dependence on skills and experiences of the planners for prostate cancer. Our results were in agreement with the results of these studies.

For FgAP plans, our results showed improved OAR sparing compared with the manual clinical plans. Many studies on the auto‐planning without FDVH guidance has shown improved or similar plan quality in comparison with the plans generated manually or using other automation algorithms.^16^, ^17^, ^19^, ^21^, ^22^ Nonetheless, the auto‐planning algorithm required users to set planning dose goals, thus introducing subjectivity. Based on the skills and preferences of the planners, the planning dose goals entered by planners might be too loose or too stringent. This study, therefore, filled this gap, providing the realistic planning dose objectives using the FDVHs that are based on dose gradients and intrinsic dose distributions. Previously, Ouyang et al.^22^ evaluated auto‐planning against the manual planning for HN cases without the feasibility guidance. The study concluded that not all OARs achieved the same feasibility level because of the different preferences of OAR sparing during plan optimization. An example of optimization technique described in Table 2 for VMAT planning showed the range of *f*‐value to derive the patient‐specific dose objectives and goals set for the optimizer.

The target dose coverage for all plans was very similar. The CIs were slightly better for FgAP plans, whereas HIs were better for clinical plans, but not significantly different. In this work, the calculations for CI and HI were performed in‐line with our institutional practice. Our institution adopted the definition of CI from International Commission on Radiation Units and Measurements Report 62 while requiring >95% of PTV receiving the prescription dose.

It is known that there exists an exponential trade‐off between dose homogeneity and OAR sparing.^27^ The median OAR doses for FgAP plans were similar to (*p* > 0.05) or lower (*p* < 0.05) than those of the clinical plans. Our results (Table 3) showed that the doses were reduced significantly for OARs located medially, such as the spinal cord, larynx, trachea, and esophagus. These findings are consistent with similar studies using FDVH‐guided HN planning by Cilla et al.,^23^, ^28^ Alves et al.,^25^ and Perumal et al.^24^ Scaggion et al.^4^ reported that the knowledge‐based DVH predictions improved the treatment plan quality and reduced the variability due to the experience of the planners. The better dose sparing for these OARs in FgAP plans resulted from faster dose falloff than those from the clinical plans as shown in Figures 3 and 4. In general, it was found that a smaller *f*‐max value corresponds to a lower OAR dose. Dose reduction to the critical OARs such as the spinal cord is important, particularly for patients who may need re‐irradiation later.

Optimal OAR sparing is desirable for all HN cancer sites especially for oropharyngeal cancer. Most of these patients have had the human papilloma virus and may have much better prognosis than other HN cancer patients. Thus, reducing normal tissue toxicity is particularly important for their long‐term quality of life. The recurrent rate of HN squamous cell carcinoma is about 25%^29^, ^30^ and re‐irradiation is the treatment choice for unresectable tumors.^31^, ^32^, ^33^ The treatment dose >40 Gy for re‐irradiation is typically prescribed; acute and/or late toxicities related to myelopathy, dysphagia, acute mucositis, trismus, carotid blowout, and osteoradionecrosis (ORN) have been correlated with the total composite dose.^29^, ^34^ Recently, the overall acute and late toxicity rates of 26% and 51% were reported, respectively, for grade ≥3 toxicity with higher incidence of trismus.^29^ Langendijk et al.^31^ reported the acute mucositis and dysphagia in a considerable proportion of patients after the re‐irradiation of oropharyngeal cancers. It was predicted that ORN and carotid blowout occur at a near maximum composite dose of 119 Gy^29^ received by the mandible and carotid arteries. Myelopathy was not frequently reported in recent publications due to the lower cumulative maximum dose of 34 Gy to the spinal cord.^29^ These findings highlight the importance of FDVH‐based predictions to spare OARs as much as possible in the initial treatment, especially for the spinal cord so that a re‐irradiation can be safely admitted if necessary.

In contrast to the KBP, the PlanIQ feasibility does not require developing and training a model based on the quality of previous plans. Kaderka et al.^15^ demonstrated non‐inferiority of plan quality of KBP‐driven HN plans to that of the manual plans. The reported doses to most of the OARs in KBP‐driven HN plans were higher than the respective OAR doses in our study. In contrast, the FDVH predictions are based on the “benchmark dose” calculated from first principles, which can be applied to unusual cases readily. It may be a useful strategy to utilize the FDVH tool to rank plan qualities prior to building and training a knowledge‐based model.^35^

The FgAP plans yielded greater MUs by 18.3% ± 18.4% on average than the clinical plans, implying greater fluence modulation and plan complexity. However, all tested FgAP plans were delivered and had gamma passing rates similar to the clinical plans.

An important concern is the planning time for VMAT plans in a busy clinic. This study was conducted retrospectively, and therefore it was not possible to compare the planning and optimization times between the clinical and FgAP plans. However, many investigators have evaluated the VMAT planning times for the Pinnacle auto‐planning module. Cilla et al.^28^ showed that the average planning time with the auto‐planning (Pinnacle 16.0 and without feasibility guidance) was reduced to 80 ± 6 min for HN plans. In their recent study on HN plan quality using the Pinnacle personalized planning (v. 16.4), they reported an average treatment planning time of 25 ± 3 min. Xia et al.^26^ reported a planning time of 33.2 ± 4.8 min for FgAP of lung plans. In general, the auto‐planning module runs six cycles of optimization with a set number of iterations in each cycle. The planning time depends on the computational power, number of beams, number of iterations, dose calculation resolution, and dose grid size. Using three VMAT arcs in HN plans and a moderate number of iterations (∼50), the first run of auto‐planning takes 60–70 min. Further post‐optimization tuning with 25–30 iterations may take additional 15–20 min for each round.^28^ The PlanIQ feasibility tool is integrated into Pinnacle v. 16.2 to facilitate planning. Thus, for this version, an optimized plan may be achieved in ∼1.5 h.

This study has several limitations. Significant contouring differences between the main campus and regional centers may alter the results of this planning study. The relative differences in experience and expertise between the planners at the main campus and regional centers may also alter the results of this planning study. This study was limited to only one treatment site, namely, HN cancer. A limited number of plans were used for the FDVH‐guided auto‐planning. Although HN cancer is one of the most complex treatment sites, it will be interesting to see how FDVH‐guided auto‐planning is applicable to other treatment sites.

## 5 CONCLUSION

This study evaluated an FDVH prediction tool to objectively evaluate the intercampus plan quality variability in treatment plans for HN cancers. This tool proved to be simple and robust. Large intercampus variations in treatment plan quality were observed in OARs, such as the spinal cord, larynx, supraglottis, trachea, and esophagus. Furthermore, the FDVH‐guided auto‐planning technique was proposed for HN plans. The FDVH‐guided plans achieved similar or better OAR sparing, particular for OARs located medially and close to the target volumes, such as the brainstem, spinal cord, esophagus, larynx, supraglottis, and trachea. The dose reductions to these critical structures would improve patient's quality of life and make re‐treatment feasible if needed. The clinical implementation of this method would be useful to reduce variability in treatment planning while generating treatment plans faster with similar or superior plan quality.

## AUTHOR CONTRIBUTION

Conception and design of study by Saeed Ahmed and Ping Xia. Patient selection by Saeed Ahmed, Nikhil Joshi, Neil Woody, and Shlomo Koyfman. Data collection by Saeed Ahmed, Chieh‐Wen Liu, Eric Murray, and Danielle LaHurd. Data analysis by Saeed Ahmed and Chieh‐Wen Liu. Manuscript preparation by Saeed Ahmed, Ping Xia, Nikhil Joshi, Matthew Kolar, Danielle LaHurd, and Chieh‐Wen Liu.

## CONFLICT OF INTEREST

PX received research grant from Philips Healthcare. Other authors have no conflict of interest related to this work.
